# Too Many Colleges

**Published:** 1896-10

**Authors:** 


### TOO MANY COLLEGES.

The *Tri-State Medical Journal*, St. Louis, has published some interesting data relative to the ratio between medical colleges and the population of the cities in which they are located. In cities having a population of less than 50,000, there is one college in Mobile (35,000), one each in Birmingham (27,000), Little Rock (42,000), Topeka (31,000), Sioux City (29,000), Kansas City, Kansas (40,000), Brunswick, Maine (6,000), Hanover, New Hampshire (2,000), Raleigh (15,000), Sewanee (1,200), Fort Worth (24,000), Saginaw, Michigan (44,000), and two each in Knoxville (24,000), and Keokuk, Iowa (17,000). Upon the data as presented by the *Tri-State Journal*, the *Lancet-Clinic* very properly remarks:

"If there is any one thing that the medical profession has been thoroughly convinced of during the past quarter of a century, it is the fact that in acquiring a medical education, clinic and anatomical facilities are absolutely essential, and such facilities to any extent are utterly impossible where there is a lack of population from which to draw material.

"Scan the list a moment, and observe a medical college in a town of 1,200, a place not large enough to support more than two physicians, who in the very nature of things cannot constitute a medical college faculty, and if every individual in the town were paupers would not furnish a modicum of clinic and anatomical material for teaching purposes.

Dartmouth, with its 2,000 population, largely constituted of college students, is a summer school. The faculty is made up of professors, who spend their vacations in the college village. The entire State of New Hampshire has not in it a sufficiency of clinic material for a medical college.

"So on, each one may be taken up in turn, and it will be found that local conditions are not such as to warrant the existence of a medical college in a single one of the cities and towns in the entire list.

"What may, and no doubt should, exist in some, but not in all of the towns in the list, is a preparatory medical school, where the two first years of the student's curriculum could be undertaken and carried on with manifest advantage. In those two years the student is not required to take up clinic study, except in the most incidental manner.

"From a considerable opportunity for observation, the writer is convinced that it requires a city population ratio of not less than fifty thousand to support a medical school. First of all, it requires that many people to support a corps of physicians from which an acceptable medical college faculty can be drawn, and even then the probability is that some of the chairs would be filled with inferior timber.

"The lesson to be learned from the above table is that there are entirely too many medical colleges, that there is no demand for a creation of new colleges; while in some flagrant instances consolidations of existing schools should be brought about.

"Two snares and delusions in medical education in the United States have been what are known as spring and summer schools, and sundown colleges.

"In regard to the former, they were created for the special convenience of those who wanted to take short cuts to graduation, *i. e.*, a young man matriculating in a fall and winter school would matriculate, take out his tickets, be enrolled, perhaps attend lectures until the holidays, but even this sometimes shirked, he having all of his tickets. In January he would hie himself off to a spring and summer school, again matriculate, obtain his lecture tickets, so that in eight months he would get in two annual courses of lectures, the latter given in two different years. The same process entered upon in his second fall of study enabled him to graduate within a year and a half, ostensibly attending three courses of lectures apparently in three years.

"Men of medicine, and particularly members of State boards of examiners should have their attention directed to this iniquitous system."

